# Concomitant Temozolomide plus radiotherapy for high-grade and recurrent meningioma: a retrospective chart review

**DOI:** 10.1186/s12885-022-09340-7

**Published:** 2022-04-07

**Authors:** Katherine Belanger, Timothy H. Ung, Denise Damek, Kevin O. Lillehei, D. Ryan Ormond

**Affiliations:** 1 Department of Neurosurgery, University of Colorado School of Medicine, Aurora, CO USA; 2 Department of Neurology, University of Colorado School of Medicine, Aurora, CO USA

**Keywords:** Meningioma, Temozolomide, Chemotherapy, Radiation Therapy, Local Recurrence

## Abstract

**Background:**

High-grade and recurrent meningiomas are often treatment resistant and pose a therapeutic challenge after surgical and radiation therapy (RT) failure. Temozolomide (TMZ) is a DNA alkylating agent that appears to have a radiosensitizing effect when used in combination with RT and may be worthwhile in meningioma treatment. Thus, we investigated the potential efficacy of concomitant RT plus TMZ compared to historical controls of just RT used in the treatment of high-grade and recurrent meningiomas.

**Methods:**

We performed a retrospective analysis of patients with meningioma treated at the University of Colorado with TMZ chemoradiation. Progression free survival (PFS) and overall survival (OS) were calculated from the start of chemoradiation to local recurrence or death, respectively.

**Results:**

Eleven patients (12 tumors) were treated with chemoradiation with a median follow-up of 41.5 months. There were two WHO grade 1, eight grade 2 and two grade 3 meningiomas. Three patients died during the follow-up period—one being disease related (11.1%). Two patients had meningioma recurrence—at 2.3 months (WHO grade 3), and 5.4 years (WHO grade 2). Three-year OS and PFS for grade 2 meningiomas were each 88%. Historical controls demonstrate a 3-year median OS and PFS of 83% and 75.8%, respectively.

**Conclusions:**

Treatment options are limited for meningiomas after local failure. In this study, TMZ chemoradiation demonstrated no significant difference in PFS and OS in the treatment of grade 2 meningiomas compared to historic controls. Further study is warranted to find novel methods for the treatment of malignant and recurrent meningiomas.

## Introduction

Meningiomas comprise about 30% of all intracranial tumors making them the most common primary intracranial neoplasm of middle to late adulthood [1–5]. 80–90% of meningiomas are classified as benign (WHO grade 1) and surgical excision is a common first line treatment [1]. Gross total resection (GTR) can be curative and is often an obtainable objective due to their circumscribed nature depending largely on location and involvement of surrounding structures [2–7]. In contrast, the other 10–20% of meningiomas are either classified as WHO grade 2 (atypical) or 3 (anaplastic) due to histologic features or local invasion of surrounding brain parenchyma [2, 7]. Higher grade meningiomas carry a much higher risk of recurrence, and quite frequently adjuvant treatment is recommended. Unfortunately, there is limited data supporting any systemic therapy options for progressive and recurrent meningioma, with no current systemic therapy guidelines [2–4]. While radiation can be a primary treatment paradigm for meningioma, it also is commonly used as adjuvant therapy after surgery and is often recommended for grade 3 tumors even after GTR and following any partial resection of grade 1 or 2 meningiomas [3, 4, 6]. Radiation is also used for treatment of recurrent disease, which can otherwise be difficult to treat. For this reason, finding novel ways to treat malignant and recurrent meningiomas remains important.

Temozolomide (TMZ) is an alkylating agent that is currently used to treat glioblastomas and brain metastases with therapeutic efficacy [8–10]. By interfering with DNA-repair enzymes, it can act synergistically with radiation therapy (RT) by making tumors more radiosensitive. Thus far, there is conflicting evidence of TMZ efficacy in meningioma treatment as a monotherapy. A prospective phase II trial resulted in none of the patients remaining progression free six months post initiation of adjuvant TMZ therapy [1]. Subsequently, two case reports have identified patients that have achieved remarkable halting of disease progression lasting over a year on adjuvant TMZ therapies [4, 11]. While these studies investigated TMZ as an adjuvant monotherapy, there is a paucity of literature on the nature of TMZ as a concomitant radiosensitizing agent for patients with recurrent/progressive meningiomas. Due to the low toxicity profile, ability to be orally administered, stable pharmacokinetics and ability to cross the blood brain barrier, TMZ is an ideal systemic chemotherapy treatment for patients with brain tumors [4, 8–10]. This study evaluated the potential efficacy of concomitant TMZ with RT as a treatment option for patients with grade 2/3 and/or recurrent meningioma.

## Methods

### Retrospective study

After obtaining IRB approval, a retrospective chart review was performed to identify all patients with a cranial meningioma diagnosis that had been treated with RT and TMZ at the University of Colorado Hospital between January 1, 2011 and May 1, 2019. Eligible patients had to be treated with RT and concomitant TMZ for their meningioma diagnosis. Eleven patients were identified and received 75 mg/m^2^/day. All research involving human participants was in accordance with the ethical standards of the institutional and national research committee and with the 1964 Helsinki Declaration and its later amendments or comparable ethical standards.

Electronic medical records and radiological imaging were accessed to identify patient demographics, histological grading and treatment course. Additionally, adverse events data were collected for TMZ. Progression free survival (PFS) was defined as time from the date of the first radiation session to the date of local recurrence/progression. Local tumor recurrence was defined by increased tumor volume and/ or evidence of new growth in the same location as seen on follow-up MRI when compared to prior MR imaging. All imaging was independently reviewed by a board-certified neuroradiologist. Overall survival (OS) was defined as the first day of radiation treatment to death. PFS and OS were determined using Kaplan–Meier analysis.

### Literature review

A review of the literature was performed of papers published in the past 10 years using the key words: meningioma AND radiation therapy within PubMed. 1402 articles were identified. Articles were eliminated if they used pediatric patient populations, included spinal meningiomas or were not in English. Two hundred and nine papers remained, and abstracts were reviewed for relevance, with further elimination for absence of adjuvant RT, use of radiosurgery, or a greatly divergent fractionated dosing schedule for adjuvant radiation than our patients received. Radiation dosing outside of 40–65 Gy total, not given in fractionated dosing, and outside of 1.5–2.5 Gy per dose were considered greatly divergent. Papers were then excluded if they did not report 3- or 5-year OS or PFS data. Due to differences in the number of participants included in the various studies, analysis was weighted to calculate the aggregated PFS and OS.

Statistical analysis was performed using Prism Graph Pad Version 9.3 (San Diego, CA, USA). PFS and OS were calculated from the start of concomitant treatment to local recurrence or death, respectively. Chi-Square analysis was used to determine if there was any statistically significant difference between 3-year PFS and OS of historical controls and our patients. A p-value of ≤ 0.05 was considered statically significant.

## Results

### Demographics

**Fig. 1:**
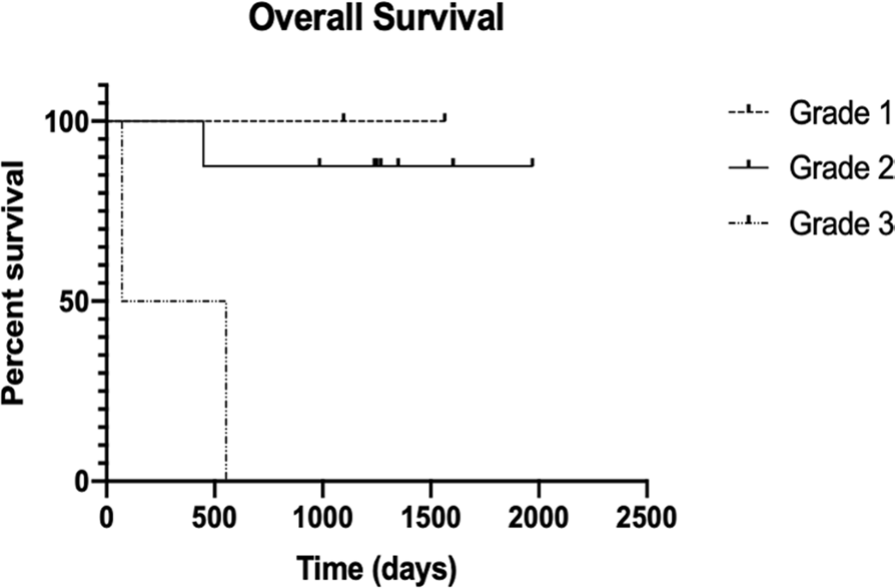
Overall Survival Curve for Grade 1, 2, and 3 Meningiomas Treated with RT and Concomitant TMZ after Initial Surgical Resection. Three of eleven patients died during the follow-up period; one being disease related at 2.3 months with a WHO Grade 3 meningioma

Eleven patients (*F* = 7, M = 4) with 12 tumors were treated with concomitant TMZ chemoradiation. The median age was 56 years (range, 22–82 years) at time of TMZ treatment initiation. All patients had at least 1 prior surgical resection of their primary tumor and received intensity-modulated radiation therapy (IMRT) with a median dose of 60 Gy (range, 48.6–65 Gy) on a 1.8–2 Gy per treatment fractionated schedule. All patients received TMZ at a dose of 75 mg/m^2^/day administered at night for 42 days. Only one patient had subsequent rounds of adjuvant TMZ for a total of six cycles taken days 1–5/28. There were two WHO grade 1, eight WHO grade 2 and two WHO grade 3 meningiomas (Table 1). The median follow-up was 41.5 months. Three of 11 patients died during the follow-up period; one being disease related from anaplastic meningioma metastasis (11.1%), and two non-meningioma related. Of the non-related deaths, one patient died from a pulmonary embolism with a known deep vein thrombosis in the setting of known cranial hemorrhages, and one patient from death from a traumatic subdural hematoma (Table 1 and Fig. 1). Two patients had meningioma recurrence during the follow-up period, one with a WHO grade 3 meningioma at 2.3 months, and one additional patient at 5.4 years (WHO grade 2).

**Table 1:** **Table 1** Patient demographics and outcome at last follow-up

WHO Grade	Age	Sex	Time to Outcome (months)	Outcome
Grade 1				
	46	F	48.1	Stable
	56	F	51.47	Stable
Grade 2				
	22	M	53.6	Stable
	57	F	11.77	Death – not due to meningioma^a^
	40	M	52.73	Stable
			41.77	Stable
	48	F	51.07	Stable
	63	F	54.9	Stable
	31	M	41.83	Stable
	70	M	64.84	Progression of Tumor
Grade 3				
	82	F	18.13	Death – not due to meningioma^b^
	59	F	2.3	Death – due to meningioma

^a^Death secondary to pulmonary embolism, known history of deep vein thrombosis in the setting of known intracranial hemorrhages

^b^Death secondary to traumatic subdural hematoma

### Literature review

Thirty-two papers, totaling 2585 patients, comprised the historical controls. The majority were retrospective papers with 12.5% being prospective. 1298 had WHO grade 1 meningiomas, 1177 WHO grade 2, and 110 WHO grade 3 [12–44]. All patients received concomitant RT after surgical resection. Sample size, weighted average, median and range were calculated for 3- and 5- year OS and PFS by meningioma grade. Only 5-year PFS and OS intervals were calculated for grade 1 meningiomas due to their relative stability and were found to have a weighted average of 87.1% and 88.8%, respectively. The weighted average PFS for grade 2 meningiomas at 3 and 5 years are 82.3% and 68.9% with weighted average OS at 3 and 5 years being 89% and 77.4%, respectively. The median 3-year OS and PFS for WHO grade 2 meningiomas was 83% and 75.8%, respectively. Weighted average PFS and OS at 3 years for grade 3 meningiomas is 28.6% and 36.2% and at 5 years it is 15.8% and 33.8%, respectively. The weighted average was calculated to take into consideration the different population sizes of each paper so that papers with a larger sample size were weighted greater than those with a smaller sample size.

### Survival analysis

**Fig. 2:**
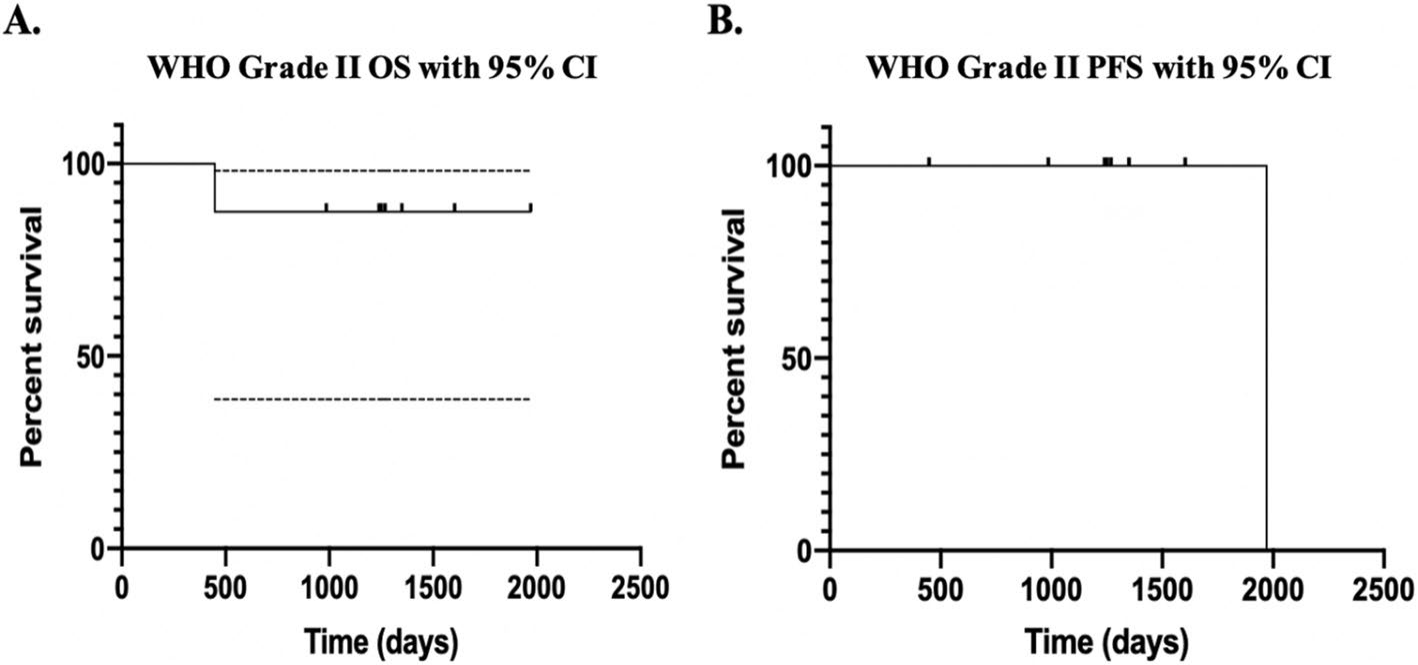
The 3-year OS and PFS for grade 2 meningiomas

Six-month PFS was 91.7%, with 83.3% remaining without local recurrence at last follow-up. The 3-year OS and PFS for grade 2 meningiomas was 88% and 88%, respectively (Fig. 2). At 3 years, only one patient with a WHO grade 2 meningioma had passed away unrelated to her meningioma less than 1 year after treatment initiation. All seven other tumors had no local progression at 3 years. No statistical difference between 3-year PFS (χ^2^ = 0.162, *p* = 0.687) and OS (χ^2^ = 0.0209, *p* = 0.885) was determined between WHO grade 2 meningiomas treated with TMZ and RT chemoradiation and historical controls of concomitant RT.

### Side effects

Of the eleven patients receiving concomitant RT and TMZ, 7 patients experienced mild fatigue, 7 patients experienced nausea, and 2 patients experienced pancytopenia. Of the seven patients with nausea, all but two cases were mild, and all patients were successfully managed with anti-emetics. One patient with severe nausea had a 10-pound weight loss secondary to the nausea and poor oral intake, requiring nutritional consultation, meal supplementation and anti-emetics. Two patients experienced hematologic toxicity: one patient had pancytopenia while the other had leukopenia and thrombocytopenia.

## Discussion

The majority of meningiomas are WHO grade 1 and thus GTR is largely curative and an obtainable objective [1–7]. High grade and recurrent meningiomas still pose a therapeutic and surgical challenge. For higher grade meningiomas, there are few treatment options and OS for these patients at 5 years is 78–91% and 35–79% for grade 2 and 3 meningiomas, respectively [5]. Our historical control data falls slightly lower than these reported ranges for 5-year OS when data is aggregated and weighted for study population size.

Currently, standard of care for high grade meningiomas and those that are sub-totally resected is adjuvant RT. Recurrent meningiomas are often treated the same with tumor debulking and adjuvant RT [3, 5, 6]. Unfortunately, recurrent tumors tend to be higher grade meningiomas and recurrent grade 2 and 3 tumors do not demonstrate proven survival benefit from adjuvant RT. Zhu et al. (2019) found no significant correlation between post-operative radiation and outcome for recurrent high grade meningiomas.

For cases that are refractory to this standard of care, there are limited systemic options, and there is a paucity of data indicating efficacy of any chemotherapeutic option. This portends poor outcomes for patients with high grade and refractory meningiomas [2–4]. TMZ has been used in patients with glioblastoma and concomitant RT plus TMZ followed by several cycles of adjuvant TMZ and demonstrates a statistically significant survival benefit when compared to RT alone [10]. In brain metastases from lung adenocarcinoma, concomitant TMZ plus RT demonstrates a benefit over RT alone [9]. Thus, there is literature showing a benefit of concomitant chemoradiation over RT alone in different CNS tumors. Due to the demonstrated benefit of TMZ as a radiosensitizing agent in other intracranial tumors, the favorable side effect profile and ease of administration, this chemotherapeutic agent may be useful in patients with high grade and recurrent meningiomas.

Our study had a PFS and OS of 88%—with one patient passing away unrelated to her meningioma about 1 year after initiation of concomitant treatment. All seven other tumors had no progression at 3 years. In comparison, the historical controls found a 3-year average and median PFS of 82.3% and 75.8% respectively and 3-year average and median OS of 89% and 83% respectively. Unfortunately, the observations of our study were not statistically significant when compared to historical controls at 3-years.

TMZ has a favorable side effect profile with the main side effects involving myelosuppression, as well as nonhematological toxicities resulting in fatigue, nausea, anorexia, vomiting and dizziness [4, 8, 9]. Seven patients experienced mild fatigue, seven experienced nausea, one of which had anorexia resulting in weight loss. These side effects were able to be sufficiently medically managed and did not alter the treatment course. Two patients had hematologic toxicities, with one patient stopping TMZ at 25 of 28 concomitant treatments, and the other patient able to complete the full chemoradiation course. Overall, TMZ was well tolerated by our patients on an outpatient basis.

Our study, and use of TMZ in other patient populations, have demonstrated the safety and tolerance of this chemotherapeutic by patients while having potential efficacy in patients with high grade and/or recurrent meningioma. Prospective clinical trials with a larger sample size are warranted to investigate the efficacy of concomitant treatment in meningioma.

### Limitations

Major limitations of this study are the short follow up period and small sample size. Meningiomas, even higher grade 2 and 3 tumors, are generally slow to develop and thus studies often have to look at longitudinal data over a decade to demonstrate median PFS or OS. Furthermore, recurrent and progressive meningioma are uncommon, making case accumulation difficult and management strategies diverse.

High grade and recurrent meningiomas demonstrate a therapeutic challenge with few efficacious options for control after primary surgical excision. Treatment of recurrent and high grade meningiomas treated with concomitant TMZ and RT is safe but treatment showed no statistically significant difference in outcome after three years in comparison to historical controls. Further study is warranted to see if there truly is a benefit to concomitant chemoradiation treatment for the management of high grade and recurrent meningiomas.

## Data Availability

The data that support the findings of this study are not publicly available due to their containing information that could compromise the privacy of research participants but are available from the corresponding author upon request.

## Abbreviations

RT: Radiation Therapy
TMZ: Temozolomide
PFS: Progression Free Survival
OS: Overall Survival
GTR: Gross Total Resection
IMRT: Intensity Modulated Radiation Therapy
